# Nanoengineering of eco-friendly silver nanoparticles using five different plant extracts and development of cost-effective phenol nanosensor

**DOI:** 10.1038/s41598-021-01609-4

**Published:** 2021-11-11

**Authors:** Siwar Jebril, Alaeddine Fdhila, Chérif Dridi

**Affiliations:** 1 NANOMISENE Laboratory, LR16CRMN01, Centre for Research on Microelectronics and Nanotechnology (CRMN), Sousse, Tunisia; 2 High School of Sciences and Technology of Hammam Sousse 4011, University of Sousse, Sousse, Tunisia

**Keywords:** Engineering, Nanoscience and technology

## Abstract

The production of environmentally friendly silver nanoparticles (AgNPs) has aroused the interest of the scientific community due to their wide applications mainly in the field of environmental pollution detection and water quality monitoring. Here, for the first time, five plant leaf extracts were used for the synthesis of AgNPs such as *Basil*, *Geranium*, *Eucalyptus*, *Melia*, and *Ruta* by a simple and eco-friendly method*.* Stable AgNPs were obtained by adding a silver nitrate (AgNO_3_) solution with the leaves extract as reducers, stabilizers and cappers. Only, within ten minutes of reaction, the yellow mixture changed to brown due to the reduction of Ag^+^ ions to Ag atoms. The optical, structural, and morphology characteristics of synthesized AgNPs were determined using a full technique like UV–visible spectroscopy, FTIR spectrum, XRD, EDX spectroscopy, and the SEM. Thus, *Melia azedarach* was found to exhibit smaller nanoparticles (AgNPs-M), which would be interesting for electrochemical application. So, a highly sensitive electrochemical sensor based on AgNPs-M modified GCE for phenol determination in water samples was developed, indicating that the AgNPs-M displayed good electrocatalytic activity. The developed sensor showed good sensing performances: a high sensitivity, a low LOD of 0.42 µM and good stability with a lifetime of about one month, as well as a good selectivity towards BPA and CC (with a deviation less than 10%) especially for nanoplastics analysis in the water contained in plastics bottles. The obtained results are repeatable and reproducible with RSDs of 5.49% and 3.18% respectively. Besides, our developed sensor was successfully applied for the determination of phenol in tap and mineral water samples. The proposed new approach is highly recommended to develop a simple, cost effective, ecofriendly, and highly sensitive sensor for the electrochemical detection of phenol which can further broaden the applications of green silver NPs.

## Introduction

Nowadays, the most research-active field of modern materials science is definitely nanoengineering and nanotechnology. An estimate made in 2020 indicates that the nanomaterials industrial production has risen to 58,000 tons per year^1^. On this basis, several methods were used for the synthesis of nanomaterials and so nanoparticles, including the physical and chemical ones^2^. However, all these methods are expensive, time-consuming, complicated, and environmentally toxic^3,4^. Furthermore, both physical and chemical methods have some limitations to control the shape and size of nanoparticles^5^. To overcome the shortcomings of these methods, biological routes have emerged as viable options. Thus, the biological approach for the synthesis of nanoparticles, which is less expensive and uses environmentally friendly resources produces non-toxic waste and with a high yield of nanoparticles would be ideal that any researcher in nanoparticles synthesis aims to achieve^6^. This is why green nanotechnology has recently found great scope in the synthesis of many nanomaterials using eco-friendly technologies rather than toxic chemicals. It has been used for the synthesis of mono and multi-metallic nanoparticles based alloys^7–10^. However, in our work we focus on simple monometallic nanoparticles. The green synthesis of metallic nanoparticles, particularly the silver ones using plant extracts as nano-factories becomes a significant subject of researches, which makes them suitable for a variety of applications, notably nano-devices and nanobiotechnology^11,12^.

The plant extracts^13–17^ and microorganisms^18,19^ such as viruses, bacteria, and yeasts play an important role in the synthesis of nanoparticles as reducers and stabilizing agents. However, there are many advantages to using the plant extracts over microorganisms, namely that they are readily available, safe to handle, they have low cultivation cost and short manufacturing time. Plants benefit also from a wide variety of metabolites such as antioxidants, proteins, vitamins, alkaloids, terpenoids, flavonoids, and phenolic compounds that play a key role in the reduction of stabilized metallic nanoparticles^20,21^. So green chemistry for plant extracts is a better option for AgNPs production cause besides their biocompatibility, stability and biodegradability, they offer a stable protection layer for the AgNPs that protects them from aggregation^22^.

The characteristics of these NPs obtained from plant extracts are influenced by different parameters such as temperature, pH and reaction time; however, the nature of the biomolecules which present in the plant extracts might be the most relevant factor in the synthesis process^23^.

For the synthesis of NPs, the primary plant compounds are responsible for the reduction of metal ions^24^, this is why we have chosen plants that provide us with a relatively fast reduction of metal ions, which easily occurs in solution and leads to a high density of stable silver nanoparticles. So, in the present work, for the first time, we report the synthesis of AgNPs exploiting five plant leaves extracts such as *Basil* (AgNPs-B), *Geranium* (AgNPs-G), *Eucalyptus* (AgNPs-E), *Melia* (AgNPs-M), and *Ruta* (AgNPs-R) as capping and reducing agents in a simple and faster process. The optical, structural, and morphology properties of these biosynthesized AgNPs were fully characterized using different techniques such as UV/visible, FTIR, XRD, EDX, and SEM analysis, resulting that the AgNPs-M have less size compared to the other types of nanoparticles, which would be interesting for phenol electrochemical sensors.

Phenolic compounds are the most polluted component that exists in the environment, we found theme in canned food, in several chemicals and in water particularly. These compounds degrade slowly in the environment and are easily absorbed through the skin or mucous membranes. Their toxicity affects a large number of organs such as the genitourinary system, the lungs, the liver, as well as kidneys. Among these phenolic compounds, phenol is the most toxic and classified as a priority pollutant^25^. Phenol exposure can cause movement problems that may include tremors, loss of coordination, muscle weakness and even paralysis. It also affects the respiratory system and can cause breathing arrest^26^. Hence, the World Health Organization (WHO) set a dose limitation of phenols not exceed 1 mg L^−1^ in drinking water^27^. For all these reasons, great efforts have been made to develop an efficient techniques for phenol determination such as spectrophotometric^28^, flow-injection analysis, chromatographic techniques^29^, and high-performance liquid chromatography (HPLC)^30^. Nevertheless, these methods are expensive, difficult to control, and time-consuming. On the other hand, electrochemical methods are lately recognized as one of the most valuable techniques for phenol detection due to their simplicity, reduced cost, and high sensing performances^31–33^, essentially when the electrode surface is modified to improve the sensitivity and the selectivity of the sensors^34^. AgNPs are among the most attractive electrochemical sensor modifier candidates. Recently, AgNPs have been used individually for the determination of the antioxidant capacity of some products with an optical sensor^35^, however, in the electrochemical detection, it has always been combined with other composites such as multi-walled carbon nanotubes (MWCNT)^36^ or polyvinyl alcohol (PVA)^37^, etc. In this work, and for the first time in our knowledge, a high sensitive, eco-friendly and cost-effective phenol electrochemical sensor, based only on biosynthesized AgNPs-M modified GCE was developed.

## Results and discussion

### Silver nanoparticles characterizations

#### Silver nanoparticles formation

**Figure 1:**
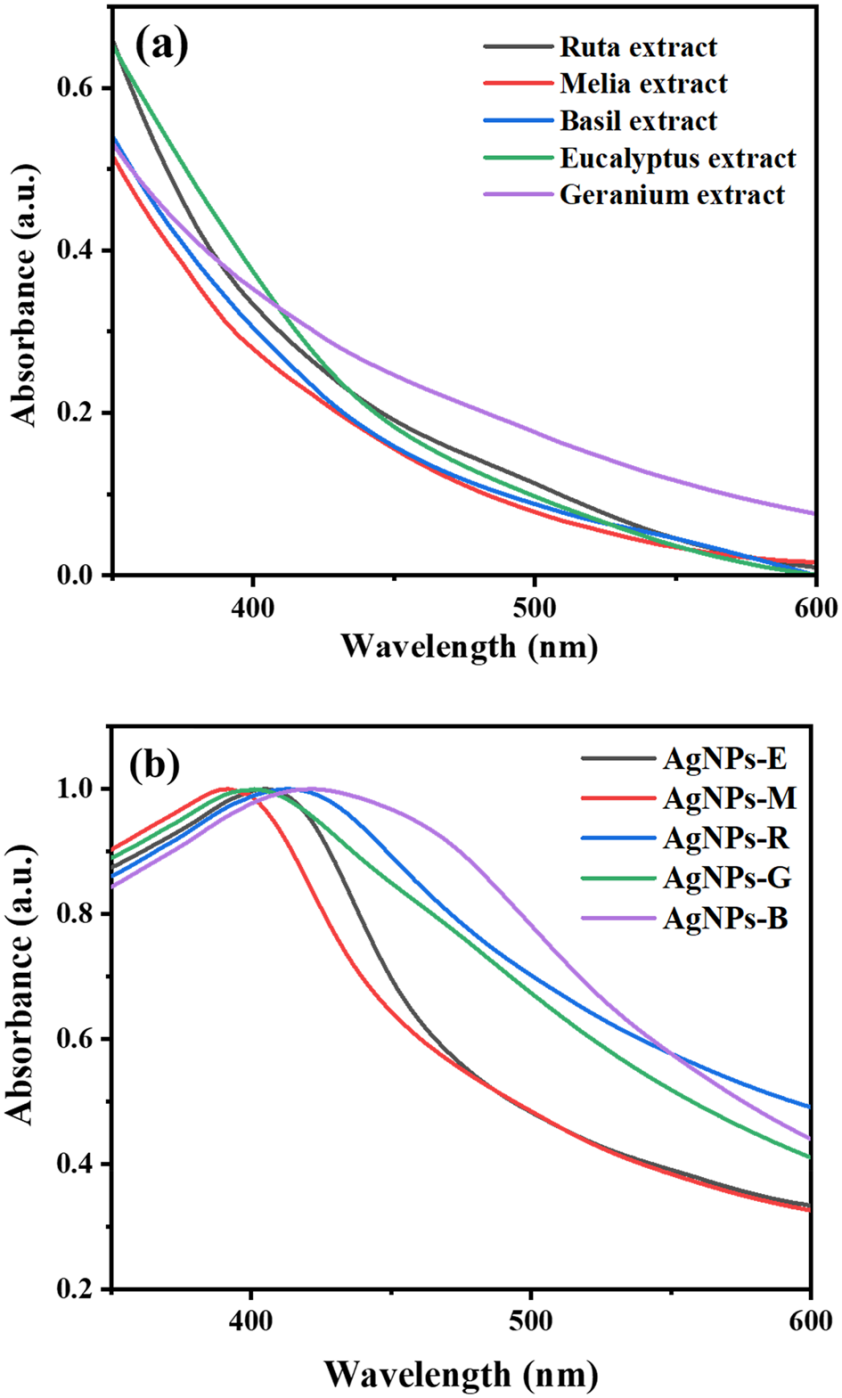
UV–visible absorption spectra (normalized) of: (**a**) leaf extracts of *Eucalyptus*, *Melia*, *Ruta*, *Geranium*, and *Basil*; (**b**) biosynthesized AgNPs.

UV–visible analysis was employed to validate the synthesized silver NPs formation through the surface plasmon resonance (SPR). Herein, AgNPs were synthesized using five different plant leaves extracts. Plants biomaterials were used as reducers, stabilizers and cappers. After 10 min, and after adding the different leaf extracts to the AgNO_3_ solution, a modification of the obtained mixture, which passes from the yellow color to the brown color, was observed, as a result of surface plasmon vibrations; this index indicates the production of AgNPs. The same color changes were obtained in other works^38,39^. The active molecules present in the leaf extracts reduce the Ag^+^ ions into Ag atoms. The NPs are then generated following the formation of clusters by the Ag atoms, and the biomolecules, present in the mixture, are responsible for their stabilization. Figure 1 illustrates the UV–visible spectrum of the leaf extracts of *Eucalyptus*, *Melia*, *Ruta*, *Geranium* and *Basil* (Fig. 1a), and the biosynthesized AgNPs (Fig. 1b). It can be seen that the UV–visible spectrum of all the leaf extracts have no absorption peak. However, after the addition of AgNO_3_ we obtained a strong absorption band at 405, 400, 415, 401, and 422 nm for the AgNPs synthesized from the leaves of *Eucalyptus*, *Melia*, *Ruta*, *Geranium*, and *Basil* respectively, due to the excitation of the SPR of AgNPs. In this study, the synthesis of AgNPs is approved by UV–visible spectrum in the range of 400–422 nm which is the characteristic wavelength range of silver nanoparticles^40^.

#### FTIR analysis

**Figure 2:**
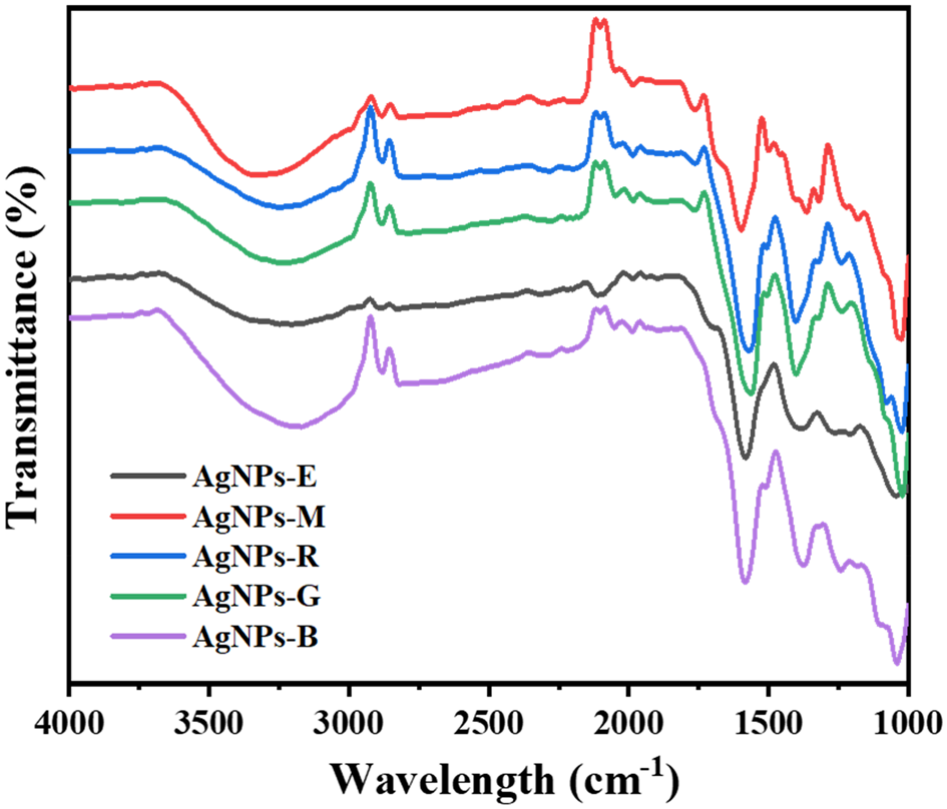
FTIR spectra of AgNPs biosynthesized using the leaf extracts of *Eucalyptus*, *Melia*, *Ruta*, *Geranium*, and *Basil* (with a shift of the transmittance axis for a 10% space between spectra for clarity of results).

**Figure 3:**
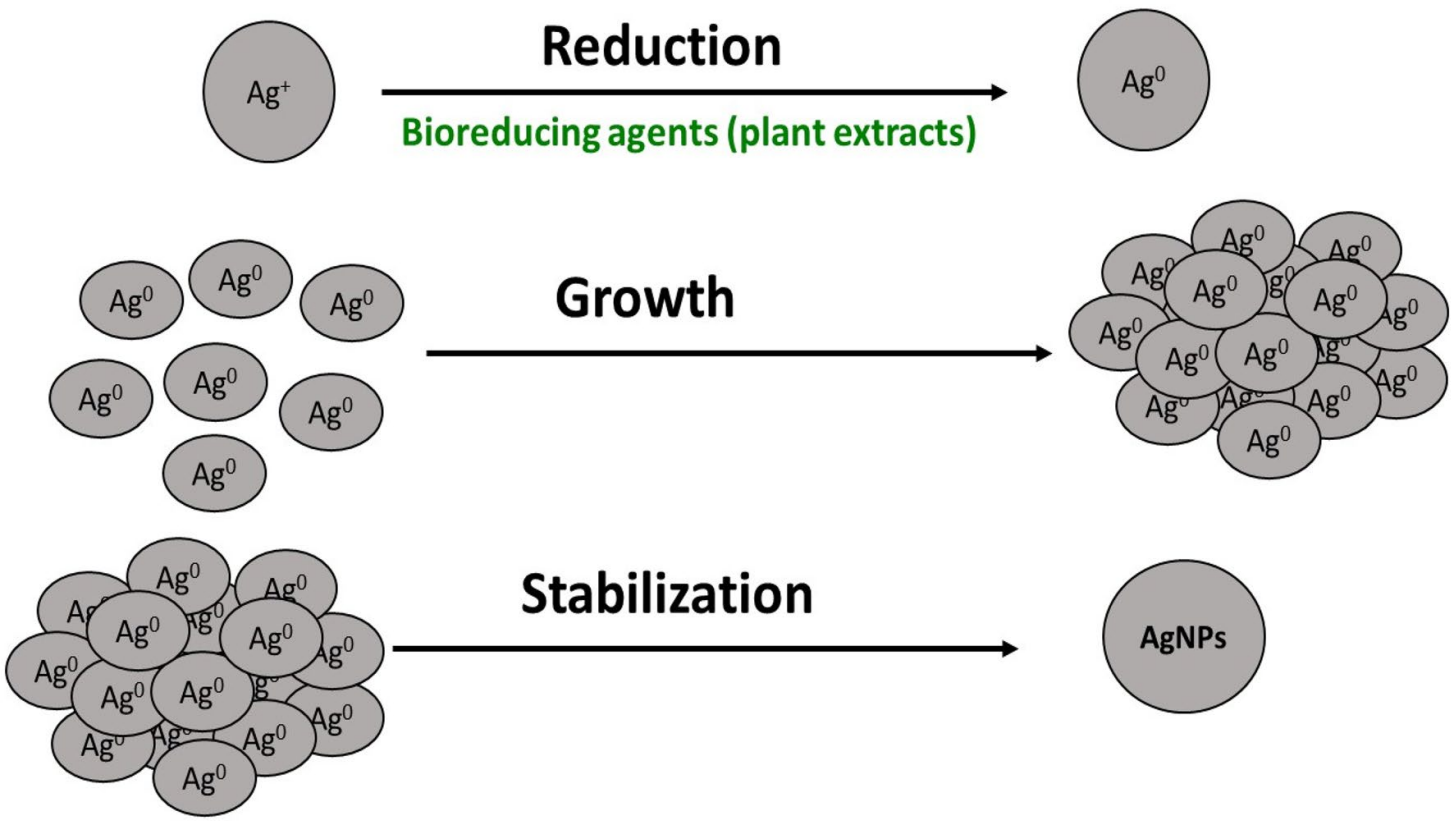
Proposed mechanism of the synthesis of AgNPs using *plant extracts*.

FTIR help us investigating the NPs functional groups and determining the biomolecules that lead to the formation of AgNPs. Figure 2 shows the FTIR spectrum of AgNPs synthesized from different leaf extracts. The large band at 2800–3500 cm^−1^ is attributed to the stretching vibration of N–H^41^. The bands observed between 1702 and 1730 cm^−1^, and 1570–1611 cm^−1^ are related respectively, to the –C=O group and amide I band of protein^42^. Then, the two other bands between 1383 and 1447 cm^−1^ and 1023–1087 cm^−1^ are characteristic of the stretching vibrations of C–N, respectively, for aromatic and aliphatic amines. It has been shown previously that proteins can be linked to NPs via free amine groups or cysteine residues in proteins. This leads to the suggestion that the protein could be the reducing and stabilizing agent of the AgNPs^43^. Figure 3 shows the mechanism suggested for the biosynthesis of AgNPs using a leaf extract.

#### XRD analysis

**Figure 4:**
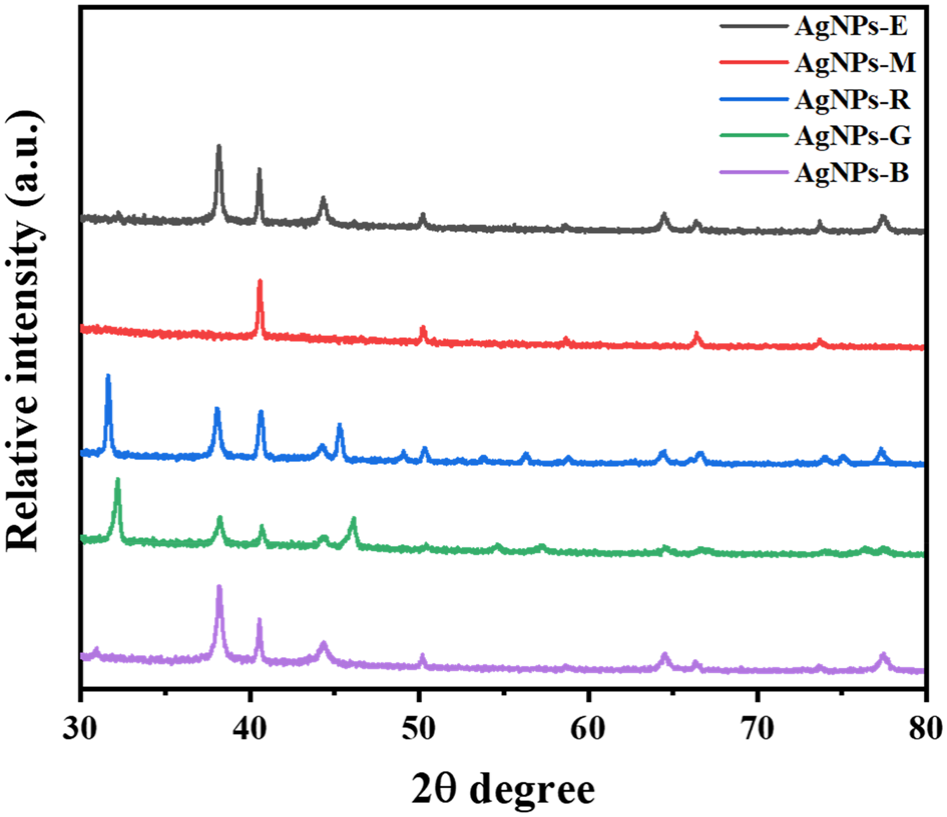
XRD pattern of AgNPs biosynthesized using the leaf extracts of *Eucalyptus*, *Melia*, *Ruta*, *Geranium*, and *Basil*.

XRD analysis was used to investigate the crystalline AgNPs structure. So, the vacuum-dried nanoparticles were reported to be elemental silver. As displayed in Fig. 4, the Bragg’s reflections showing (111), (200), (220), and (311) plans of FCC crystalline structure due to metallic silver, are presented at the range of 38°–41°, 48°–51°, 65°–67°, and 72°–75°, respectively. The AgNPs average size was calculated using the Scherrer formula, as mentioned elsewhere^40^. The size of the different types of NPs was around 40, 30, 50, 21, 26 nm for AgNPs-B, AgNPs-G, AgNPs-E, AgNPs-M, and AgNPs-R, respectively. It has been reported that the electrocatalytic activity of NPs is highly dependent on their size and their morphology^44,45^. Besides, the presence of smaller nanoparticles could be beneficial for sensor development than the larger ones. Here, AgNPs-M is smaller in size than other types of nanoparticles like AgNPs-B, AgNPs-G, AgNPs-R, and AgNPs-E.

In this perspective, AgNPs-M seems to be the most suitable for the electrochemical study, due to its small size, and hence its use in our following work.

### Application of AgNPs-M for the development of phenol sensors

#### Nanostructural properties: SEM and zeta sizer studies

**Figure 5:**
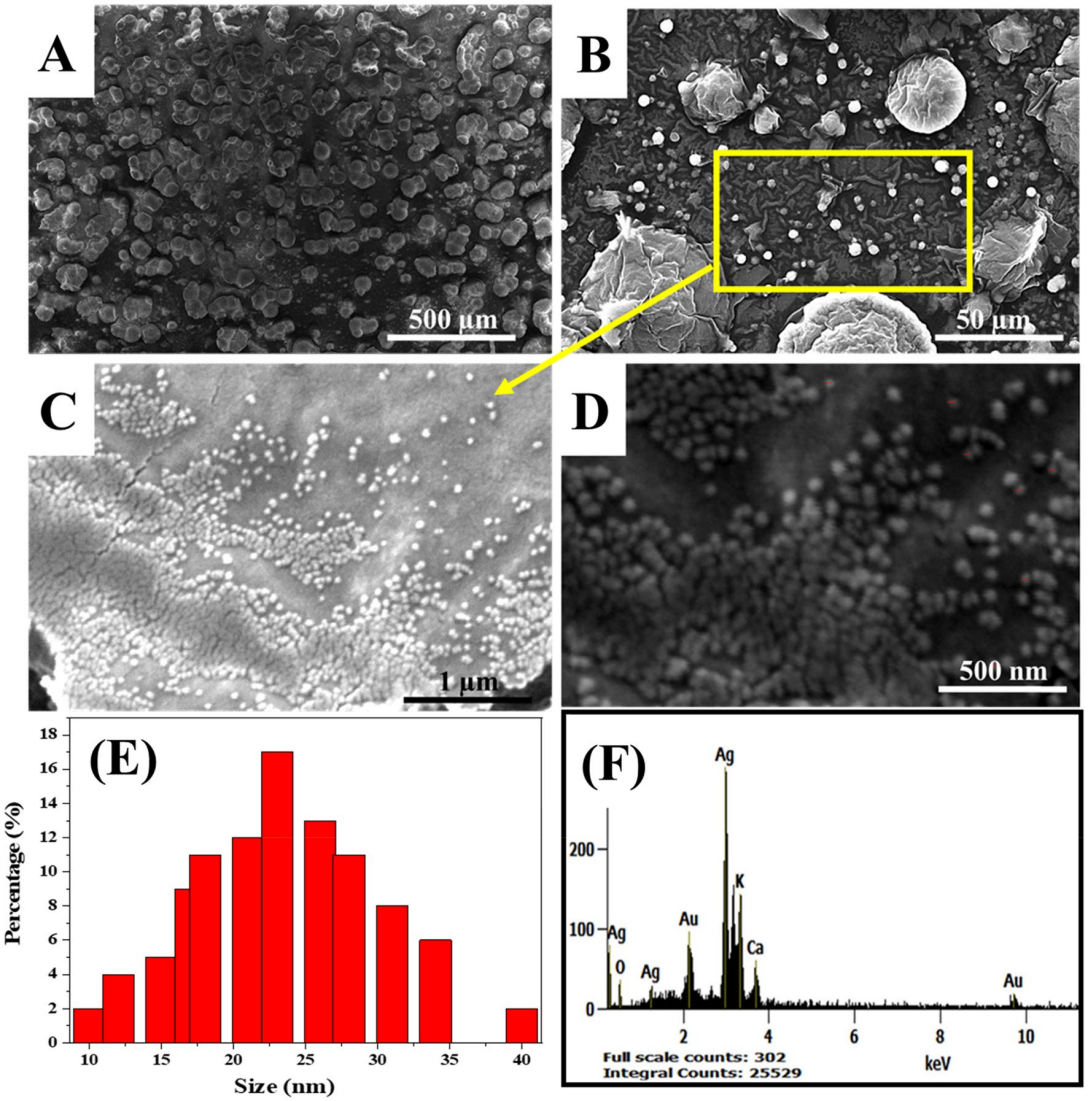
(**A**–**D**) SEM images of AgNPs-M at different magnifications: (**A**) ×100, (**B**) ×1000, (**C**) ×50,000, (**D**) ×100,000, (**E**) a histogram with the size distribution of AgNPs-M, and (**F**) EDX images of AgNPs-M.

EM analysis was utilized to identify the morphology and the size of stabilized AgNPs-M at several magnifications (100×, 1000×, 50,000× and 100,000×) (Fig. 5A–D). SEM results showed that AgNPs-M has spherical morphologies and 23 ± 3 nm as an average size, with a high percentage of nanoparticles (Fig. 5E). However, we can find some bigger NPs with a low percentage of about 15%, probably due to the agglomeration of small ones. In addition, the zeta potential results of green AgNPs indicate a negative value of − 13.1 mV, which indicates the good stability and the correct dispersion of AgNPs.

Furthermore, EDX analysis was employed to define the chemical composition of AgNPs-M. As shown in Fig. 5F, at 3 keV, we notice a high signal corresponding to elemental silver, confirming the AgNPs presence^46,47^. Besides, O, K and Ca atoms signals, obtained due to the plant residues capping the AgNPs (Table 1).

**Table 1:** EDX elemental analysis of AgNPs-M.

Element	Mass (%)	Atom (%)
Ag L	56.81	27.84
Ca K	6.17	8.13
Au L	13.48	3.62
O K	14.64	48.37
K K	8.90	12.03

After green AgNPs nanostructural properties investigation, precisely the size, the stability, and the chemical composition, the results prove their utility for the development of an AgNPs-M/GCE structure based phenol nanosensor, and this is the purpose of the next section.

#### Performances study of the AgNPs-M/GCE based phenol sensor

**Figure 6:**
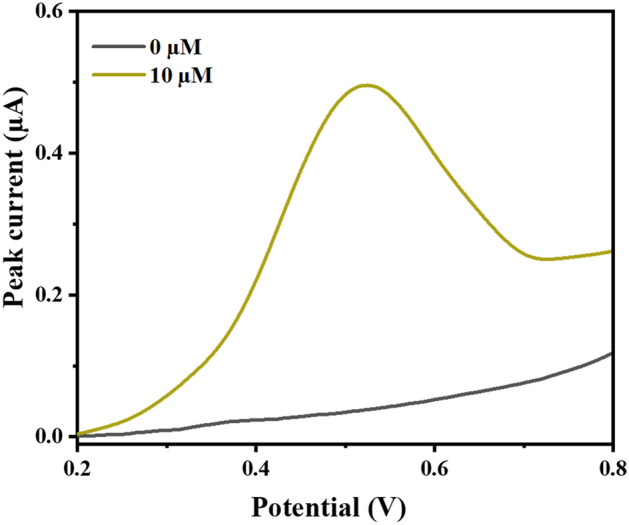
DPV voltammogram recorded at AgNPs-M/GCE for 0 µM and 10 µM of phenol concentrations.

**Figure 7:**
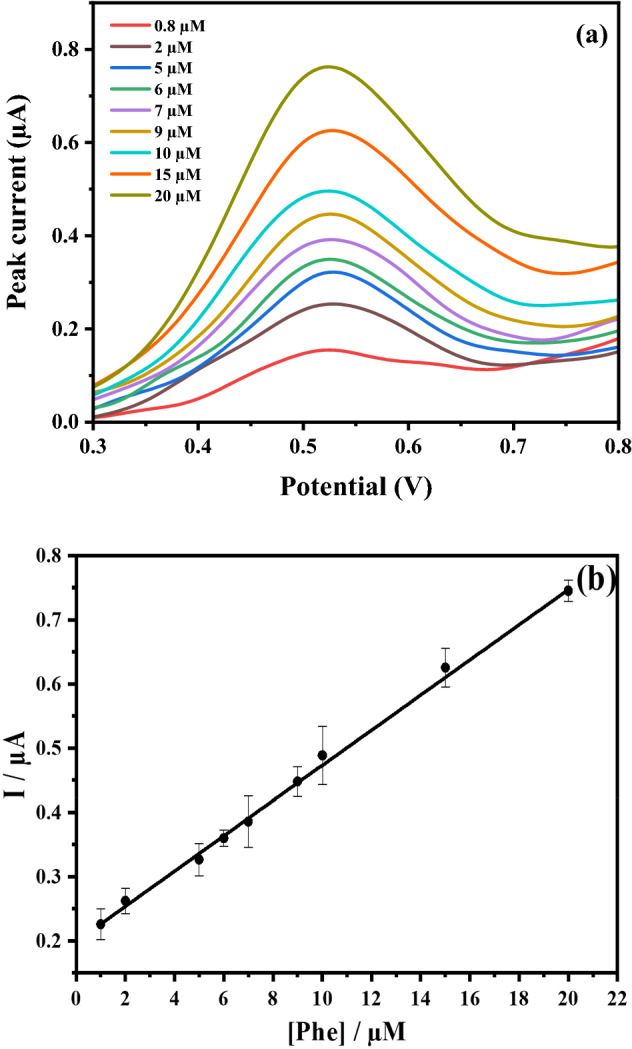
(**a**) DPV voltammogram recorded at AgNPs-M/GCE for increasing phenol concentrations (0.8, 2, 5, 6, 7, 9, 10, 15 and 20 µM) in PBS (0.1 M, pH 7.0), at the pulse time of 100 ms, pulse amplitude of 100 mV and at the scan rate of 60 mV s^−1^; (**b**) corresponding calibration curve.

The electrochemical properties of AgNPs-M synthesized from *Melia* extract were estimated by studying the electrochemical behavior of 10 µM of phenol (Fig. 6). As we can see in Fig. 7, the electrochemical determination of phenol on AgNP-M/GCE sensor in 0.1 M PBS (pH 7.0) by DPV technique and for a concentration range from 0.8 to 20 µM. The calibration curve is following the equation: *I* (µA) = 0.410 [*phenol*](µM) + 0.02 (*R*^2^ = 0.998).

We notice the high sensitivity, of about 0.410 A/M, of our developed sensor. The LOD was calculated according to the equation: (3 × *SD*_*blank*_)/*slope* was 0.42 µM (n = 3). Table 2 shows several of the LODs mentioned in the literature. We can see that our obtained LOD is one of the lowest values.

**Table 2:** Comparison of the performance of our proposed sensor with other phenol sensors.

Electrode material	Electroanalytical technique	Linear range (μM)	Limit of detection (μM)	Refs.
Poly(zincon) electrode	DPV	21–292	9	^48^
		357–922		
rGO–ZnO NPs	DPV	2–15 15–40	1.94	^49^
AgNP/MWCNT/GCE	SWV	2.4–152	3	^50^
Ni/a-MWCNT/GCE	CV	10–480	7.07	^51^
Zincon–CTAB composite/SPE	DPV	0–300	2.2	^52^
Au/CeO_2_/g-C_3_N_4_/CP	CV	10–90	2.3	^53^
MnO_2_/SPCE	Chronoamperom	10–1000	6.7	^54^
AgNPs/GCE	DPV	0.8–20	0.42	This work

*rGO* reduced graphene oxide, *ZnO* zinc oxide, *MWCNT* Multiwall carbon nanotube, *SWV* Square wave voltammetry, *a-MWCNT* acidified multiwalled carbon nanotube, *CV* Cyclic voltammetry, *CTAB* Cetyl trimethyl ammonium bromide, *SPE* Screen-printed electrode, *Au/CeO*_*2*_ Gold–cerium oxide, *g-C*_*3*_*N*_*4*_ graphite-carbon nitride, *CP* Carbon paper, *MnO*_*2*_ Manganese dioxide, *SPCE* Screen-printed carbon electrode.

#### Reproducibility, repeatability, and stability at AgNPs-M/GCE

Reproducibility, repeatability, and stability are the main elements of sensor performance. Using three different similarly prepared electrodes and in 5 μM of phenol, reproducibility was verified by DPV, showing an RSD of 3.18% (n = 3). Repeatability was tested through the response of 6 µM of phenol using the same electrode, three times in succession, showing an RSD of 5.49% (n = 3). The stability of our proposed sensor was estimated by conserving the electrode for four weeks and measuring the phenol twice a week. After this period, the sensor maintained 93.11% of its initial response for the determination of phenol, indicating good stability of AgNPs-M/GCE. The results obtained indicate the good reproducibility of our proposed sensor besides a good repeatability and long-term stability.

### Selectivity of the developed sensor towards other phenolic compounds

To estimate the selectivity of the developed phenol sensor towards other phenolic compounds (nanoplastics) such as bisphenol A (BPA) and catechol (CC), particularly for their detection in mineral water contained in plastic bottles, we have carried out their simultaneous analysis. The obtained results showed that the peak potential of phenol was quite stable with a slight decrease of the oxidation current peak by less than 10% in the presence of different concentrations of BPA and CC. Therefore, our proposed simple, cost effective and eco-friendly nanosensor exhibits good selectivity for the determination of phenol with a potential opportunity of application for its fast analysis in the water contained in plastics bottles.

### Practical applications in water analysis

To assess the response, in real applications, of the AgNPs-M/GCE structure based phenol sensor, the presence of phenol was investigated in samples of tap and mineral waters. In this context, each of these real samples was prepared by adding 5 ml of water and 5 ml of PBS, then the determination was studied by DPV under optimized experimental conditions. The first test was without phenol, then we have added 5 and 8 µM of phenol to the water sample. Table 3 shows the recovery results which are about 100% and 107.4%. These good results of our AgNPs-M/GCE based phenol nanosensor, showed that it is highly adequate for application in real water samples.

**Table 3:** Recovery percentages and errors obtained after the determination of phenol in tap and mineral water samples.

Samples	Added (μM)	Found (μM)	Recovery (%)	*RSD (%)
Mineral water	5	5.37	107.4	7
	8	8.47	105.8	6
Tap water	5	5.08	100	5.73
	8	8.23	103	4.91

*n = 3.

## Conclusions

We have demonstrated, that the synthesis of AgNPs is possible by a simple and rapid biological route using various extracts from the leaves of plants such as *Basil*, *Geranium*, *Ruta*, *Eucalyptus*, and *Melia* at room temperature. Biosynthesized nanomaterials have been well characterized by several techniques such as UV/visible, XRD and FTIR spectroscopy, and electron microscopy (SEM and EDX), resulting that the silver nanoparticles synthesized from *Melia azedarach* leaves extract (AgNPs-M) have less size at about 23 ± 3 nm compared to the other types of nanoparticles, which would be interesting for electrochemical sensors activity. The AgNPs-M were used as catalysts in electroanalysis, and were drop-casted onto the surface of Glassy Carbon transducers to build a novel AgNPs/GCE modified nanosensor. This sensor was employed for determination of phenol, obtaining excellent figures of merits concerning: high sensitivity, low LOD of 0.42 µM, good stability with a lifetime of about 1 month and also a good selectivity towards other phenolic compounds with a deviation less than 10%, as well as repeatable and reproducible results with an RSD of 5.49% and 3.18% respectively, thanks to the green AgNPs as cost-effective nanomaterials. Besides, our proposed nanosensor has been employed successfully for phenol determination in tap and mineral water samples showing a good recovery percentage values between 100 and 107.4%.

We think that our results provide an exciting perspective for sustainable large-scale nanoplastics sensors production by glassy carbon electrode surface engineering using green nanoparticles for water quality monitoring.

## Methods

### Materials

The plants such as *Basil, Geranium, Eucalyptus, Melia,* and *Ruta* were gathered from the ISA-CM (Tunisia). AgNO3 was purchased from Sigma-Aldrich, Germany. Phenol, bisphenol A (BPA), and catechol were obtained from Sigma-Aldrich. Dipotassium phosphate (K_2_HPO_4_) and monopotassium phosphate (KH_2_PO_4_) from Merck (Germany), were utilized in the preparation of the phosphate buffer solution (PBS).

### Declaration statement

We declare that the collection of plant material is in accordance with relevant institutional, national and international guidelines and legislation.

### Preparation of plant leaf extracts

**Figure 8:**
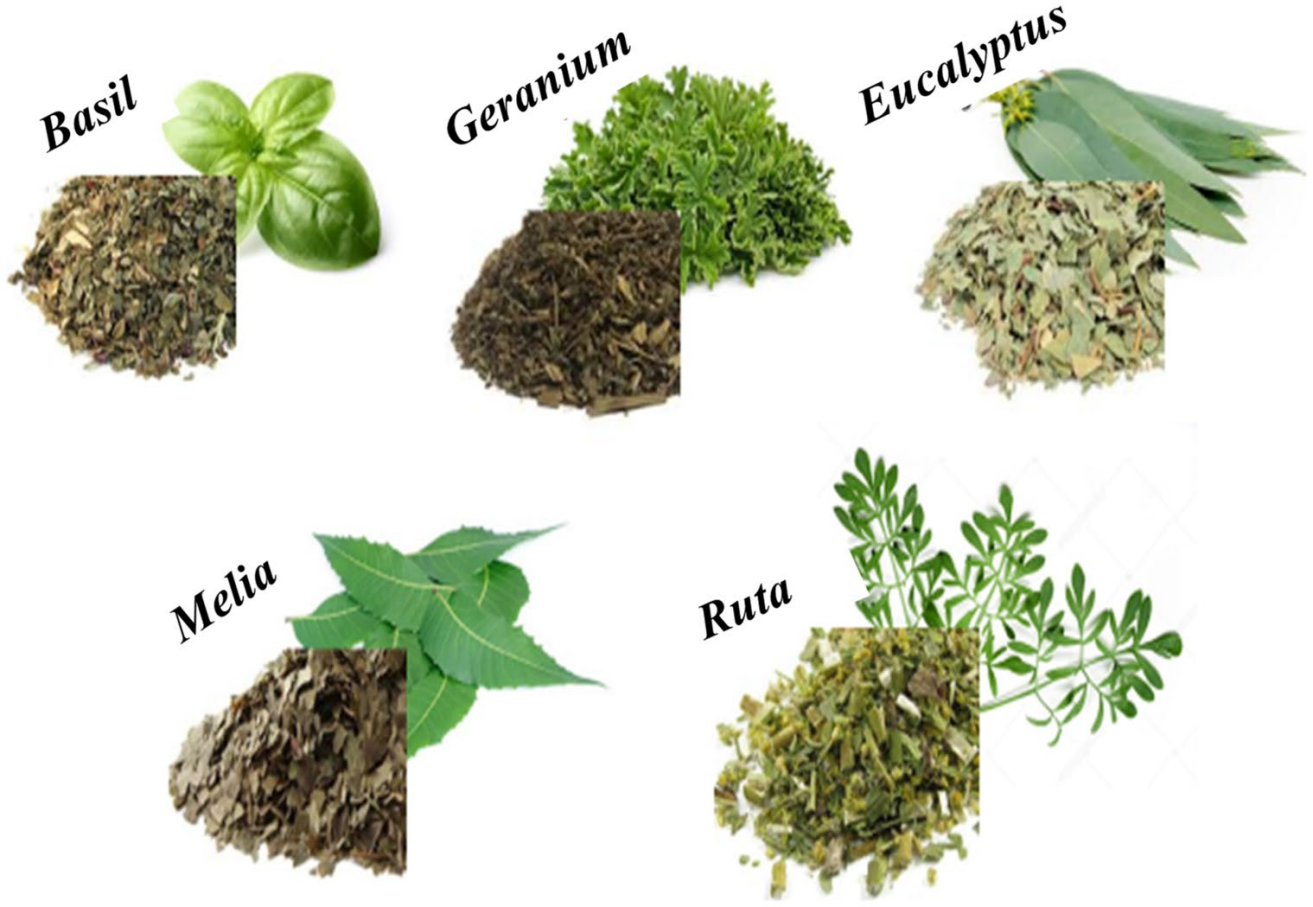
Plants used in the synthesis of AgNPs.

The plant leaves were collected and washed multiple times to eliminate all dirt particles. The cleaned leaves were parched for 12 h in an oven at 60 °C temperature and then powdered (Fig. 8). An amount of 2 g of this powder was mixed, for 5 min, with 50 ml of boiling water. Finally, the obtained solution was spun 15 min, at 5000 rpm, in a centrifuge, filtered using filter paper, and then stocked in refrigerator at 4 °C for future experiences.

### Green synthesis of AgNPs

The synthesis procedure was explained in our previous work^40^. A volume of 10 ml of 0.025 M AgNO_3_ solution was mixed with 40 ml of each aqueous plant extract. Before being filtered and dried, the mixed solution was put to heat for 10 min at 40 °C, and as evidence of the creation of AgNPs, we can see a deviation from yellow to brown. Finally, the obtained NPs were stored for further experiments.

### Instrumentation

The synthesized silver nanoparticles formation, was assured using UV–visible spectroscopy. Their optical properties were done using UV–Vis spectrophotometer Unico S-2150, from 200 to 800 nm, with 1 nm resolution.

The FTIR Spectrometer (Perkin Elmer Spectrum 100) was used at resolutions of 4 cm^−1^, between 4000 and 500 cm^−1^.

The size and crystalline structure of biosynthesized AgNPs were studied using XRD analysis by a Panalatical X’pert PRO diffractometer (PXRD) using CuKα radiation (λ = 1.54 nm) with the scanning 2θ angle in the range of 30°–80° at room temperature.

The morphologies and the average size of the AgNPs-M were obtained using a high-resolution FEI Q250 Thermo-Fisher ESEM with 2.9 nm resolution at 30 kV.

EDX was utilized to study the composition of the AgNPs-M. The detector EDX was bound with the SEM instrument.

### Fabrication of AgNPs-M/GCE based phenol sensor

**Figure 9:**
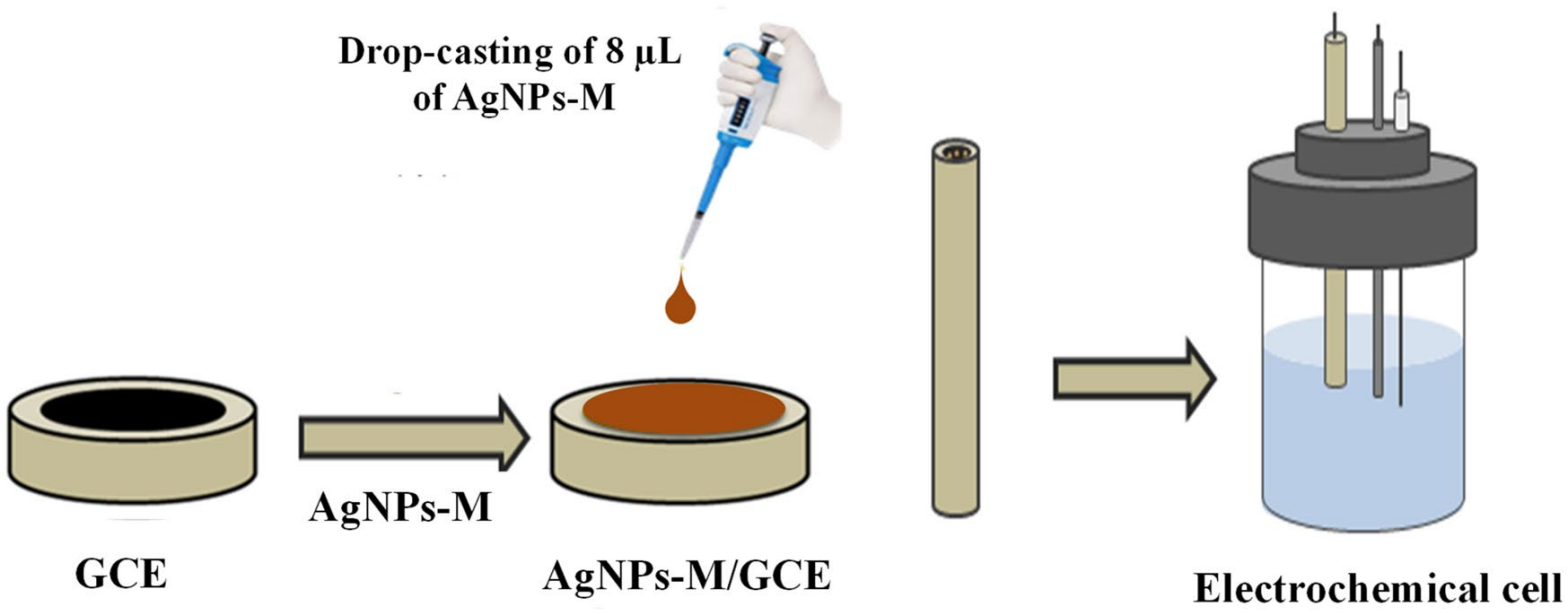
The fabrication process of AgNPs-M/GCE sensor.

The fabrication of our electrochemical sensor based on GCE modified with synthesized silver nanoparticles from a Melia leaf extract (AgNPs-M/GCE), has been carried out by casting 8 µl of AgNPs-M onto the clean surface of GCE (Fig. 9). Then, the prepared AgNPs-M/GCE was dried in darkness at ambient temperature for 24 h.

### Electrochemical measurements

The EC measurements were performed, by gradually raising the phenol concentration in the PBS solution, in an electrochemical cell containing three electrodes, the working electrode is GCE, the counter electrode is made of Pt, and the reference electrode is Ag/AgCl.

The electroanalytical technique used is the differential pulse voltammetry (DPV) in the potential range of 0.1–0.9 V and as parameters we have the PT of 100 ms, SS of 60 mV s^−1^, PA of 100 mV.

## Abbreviations

EC: Electrochemical
PA: Pulse amplitude
EDX: Energy-dispersive X-ray
Pt: Platinum
FCC: Face center cubic
PT: Pulse time
FTIR: Fourier transform infrared
SEM: Scanning electron microscopy
GCE: Glassy carbon electrodes
SS: Scan speed
NPs: Nanoparticles
XRD: X-ray diffraction
LOD: Limit of detection
